# Enhancement of Immune Responses by Co-delivery of CCL19/MIP-3beta Chemokine Plasmid With HCV Core DNA/Protein Immunization

**DOI:** 10.5812/hepatmon.14611

**Published:** 2014-03-01

**Authors:** Christine Hartoonian, Zargham Sepehrizadeh, Mojtaba Tabatabai Yazdi, Yong Suk Jang, Lida Langroudi, Parisa Amir Kalvanagh, Babak Negahdari, Ali Karami, Massoumeh Ebtekar, Kayhan Azadmanesh

**Affiliations:** 1 Department of Pharmaceutical Biotechnology and Biotechnology Research Center, Faculty of Pharmacy, Tehran University of Medical Sciences, Tehran, IR Iran; 2 Department of Virology, Pasteur Institute of Iran, Tehran, IR Iran; 3 Departments of Molecular Biology and Bioactive Material Sciences, Institute for Molecular Biology and Genetics, Chonbuk National University, Jeonju, Korea; 4 Department of Immunology, Faculty of Medical Sciences, Tarbiat Modaress University, Tehran, IR Iran; 5 Department of Medical Biotechnology, School of Advanced Technologies, Tehran University of Medical Sciences, Tehran, IR Iran; 6 Department of Research Center of Molecular Biology, Baqyiatallah University of Medical Sciences, Tehran, IR Iran

**Keywords:** Chemokine CCL19, Hepatitis C, Hepatitis C Antigens, Adjuvants

## Abstract

**Background:**

Using molecular adjuvants offers an attractive strategy to augment DNA vaccine-mediated immune responses. Several studies have revealed that an efficient HCV vaccine model should be able to induce both humoral and cell mediated immune responses targeting the conserved regions of the virus to circumvent the immune escape mutants. The beta chemokine Macrophage Inflammatory Protein 3-beta (MIP-3beta) is a key modulator of dendritic cells (DCs) and T-cells interaction, functions during immune response induction and is secreted specifically by cells in the lymphoid tissues.

**Objectives:**

In the present study, we questioned whether co-administration of MIP-3beta gene could enhance the immune responses to HCV core in DNA vaccination.

**Materials and Methods:**

Expression and biological activity of MIP-3beta expressing plasmid were evaluated by ELISA and transwell migration assays, respectively. HCV core DNA vaccine ± plasmid expressing MIP-3beta were electroporated subcutaneously to the front foot pads of BALB/c mice on days 0 and 14, and HCV core protein booster was applied to all core-DNA-vaccine received mice on the day 28. Both cell mediated immunity (proliferation, IFN-γ and IL-4 cytokine release, IFN-γ ELISpot and cytotoxic Granzyme B release assays) and humoral immune responses (total IgG and IgG2a/IgG1 subtyping) were evaluated ten days after final immunization.

**Results:**

Mice covaccinated with MIP-3beta elicited an enhanced Th1 biased systemic immune response as evidenced by higher IFN-γ/IL-4 and anti-core IgG2a/IgG1 ratio, lymphoproliferation, strong cytolytic GrzB release and enhanced population of IFN-γ producing immunocytes. Likewise, the humoral immune response assumed as the total anti-core IgG level was augmented by MIP-3beta co-delivery.

**Conclusions:**

These results exhibited the immuno potentiator effects of MIP-3beta plasmid when coadministrated with the HCV core DNA vaccine. Complimentary studies integrating MIP-3beta as a genetic adjuvant in HCV-core-DNA vaccination models are warranted.

## 1. Background

Hepatitis C virus (HCV) is a hepatotropic enveloped RNA virus belonged to the Hepacivirus genus in the Flaviviridae family (1). HCV infection is a serious health problem with an estimated 3-4 million newly infected people each year worldwide, making it the second most common chronic viral infection. It causes acute and chronic hepatitis in humans and chimpanzees (2). Chronic hepatitis C can progress to cirrhosis and hepatocellular carcinoma (HCC) causing death in more than 350,000 infected people annually (3). In some recent cohort clinical trials, triple therapy combining one of the newly FDA approved protease inhibitors (PIs) with the conventional PEG IFN-α/Ribavirin regimen have been shown to improve the sustained virologic response (SVR) rate up to 75% (4, 5); yet the need for an HCV prophylactic/therapeutic vaccine remains of vital importance. The HCV core protein is the viral nucleocapsid encoded by the 5' end of HCV genome ORF (open reading frame). It is one of the most conserved proteins among the various HCV genotypes suggesting it as a superior antigen candidate for HCV vaccine development, despite the fact that it is not known as an strong immunogen in DNA vaccination (6, 7). Since the HCV genome was first isolated in 1989 (8), efforts to develop an effective vaccine had limited success, and there is no licensed vaccination strategy available yet. DNA vaccines represent an attractive strategy against infectious diseases by induction of both humoral and cell-mediated immune responses. The major shortcoming of DNA vaccines is the poor induction of immunity (9). To overcome this obstacle, diverse strategies have been introduced including utilization of immune modulator molecules like cytokines as molecular adjuvants (10). Chemokines are small proinflammatory cytokines playing major roles in leukocyte migration and activation. Some chemokines modulate differentiation of effector T cells into Th1 and Th2 polarized phenotypes (11). Several studies have shown enhancement in immune response induction when cytokines/chemokines are included as molecular adjuvants (10, 11). Macrophage Inflammatory Protein-3beta / Epstein-Barr virus-induced-molecule-1-ligand-chemokine (MIP-3beta /ELC/CCL19) is a CC chemokine mainly expressed in secondary lymphoid organs and binds to the chemokine receptor CCR7 (12). CCR7 is expressed on mature dendritic cells (DC) and distinct T- and B-cell subpopulations (13). This chemokine plays a pivotal role in directing DCs and T-cells into lymphoid tissues and modulation of the interactions between the DCs and T cells which eventually give rise to antigen specific T lymphocytes capable of counteracting an infection or tumor (14). MIP-3beta secreted by mature DCs increases naïve T cells scanning behavior and their response to rare cognate antigens. It also lowers the threshold of activation of naïve T cells while increasing their sensitivity to low density antigen presentation (15). Furthermore, MIP-3beta has been previously reported as immunopotentiator in vaccination strategies (16-19).

## 2. Objectives

To enhance the HCV core DNA vaccine immunogenicity, the immunoadjuvant activity of MIP-3beta expressing plasmid in co-delivery with HCV core DNA vaccine was assessed in a DNA/DNA/protein immunization regimen via evaluating both humoral and cell-mediated immunities.

## 3. Materials and Methods

### 3.1. Plasmid Constructs

HCV core expressing DNA vaccine was constructed and evaluated as previously described (20). Briefly, the core region of HCV genome was amplified from the sera with positive results for genotype 1 HCV and cloned into pcDNA3 (Invitrogen, CA) (summarized as pCore here). Murine MIP-3beta cDNA cloned into pcDNA3.1 + plasmid (Invitrogen, CA) was provided by Cytokine Bank, South Korea (summarized here as pMIP-3beta). The construct integrity was confirmed by restriction enzyme mapping, PCR and DNA sequencing (data not shown). Endofree plasmid Giga Kit (Qiagen, Germany) was used to prepare plasmids in large scale. 

### 3.2. Expression and Biological Activity of MIP-3beta

**Figure 1.**
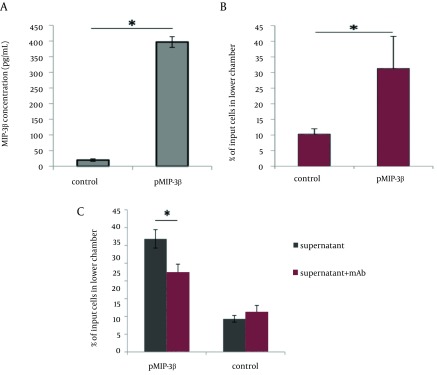
Expression and Chemotactic Activity of MIP-3beta A) Sandwich ELISA was used to confirm the expression of MIP-3beta. pcDNA 3.1+ was used as control in transfection experiments. B) Induced chemotaxis of CCR7+ HUT-78 cell line by the secreted MIP-3beta was evaluated by examining the percentage of migrated cells to the lower chamber in the presence of pMIP-3beta-transfected cell supernatant. Backbone plasmid-transfected supernatant was used as control. C) Inhibition of MIP-3beta induced chemoattraction by antibody-mediated neutralization of chemokine (mAb: MIP-3beta neutralizing monoclonal antibody). All experiments were performed in duplicate and repeated three times. Results are expressed as mean percentage of migrated cells to the lower chamber of transwell ± standard deviation (SD). Asterisks represent significant deference (p < 0.01)

HEK 293T cells (National Cell Bank of Iran) were cultured in 6-well plates (Orange Scientific, Belgium) at a density of 2.0 × 10^5^ cells/well in conditioned chemotaxis medium (DMEM supplemented with 1% BSA). The next day, cells were transfected with 2.5 µg of pMIP-3beta or pcDNA3.1+ control vector using Lipofectamine LTX transfection reagent (Invitrogen, CA) according to the manufacturer’s protocol. Cell culture supernatants were harvested after 72 h and assayed for the presence of MIP-3beta using commercial mouse CCL19 Duo Set ELISA kit (R&D systems, Minneapolis, MN) following the manufacturer’s instruction. The chemotaxis assay was performed using a microchamber-transwell system with 8 μM pores (SPL Life Sciences Inc., Korea). A total of 1 × 10^6^ cells of HUT-78 (Human T cell lymphoma expressing CCR7 chemokine receptor (21), National Cell Bank of Iran) were resuspended in 100 µl RPMI 1640 supplemented with 1% BSA and then were applied to the upper chamber, while the pMIP-3beta transfection supernatant was administered to the lower compartment of the chamber. Cells were allowed to migrate to the lower compartment for 4h at 37°C. Cells transmigrated to the lower chamber were harvested and counted with microscope using hemocytometer, and the results were expressed as percentage of migrated cells. To further determine whether the induced chemotaxis was specifically mediated by chemokine plasmid expressing MIP-3beta, supernatants of pMIP-3 beta and backbone plasmid-transfected cells were pre-incubated with CCL19/ MIP-3beta monoclonal antibody ((Clone 87102), Rat IgG2A (R&D systems, Minneapolis, MN)) with a concentration of 30 µg/mL for 20 min at room temperature to specifically neutralize MIP-3beta; then 600 µL of the mixtures were added to the transwell chambers for migration assay (Figure 1). 

### 3.3. Mice and Immunization

The injection regimen was decided following preliminary experiments (data not shown) BALB/c mice (female, aged 5-7 weeks) were purchased from Pasteur Institute (Karaj, Iran) and handled according to the criteria outlined in the "Guide for the Care and Use of Laboratory Animals" prepared by the National Academy of Sciences and published by the National Institutes of Health (NIH publication 86-23 revised 1985). DNA immunizations were performed on days 0 and 14 immediately followed by electroporation. On the day 28, HCV core protein booster was administered. Ten days after the final immunization, both humoral and cell-mediated immune responses were assessed 

### 3.4. Cellular Immune Responses

#### 3.4.1. Splenocyte Isolation

**Figure 2.**
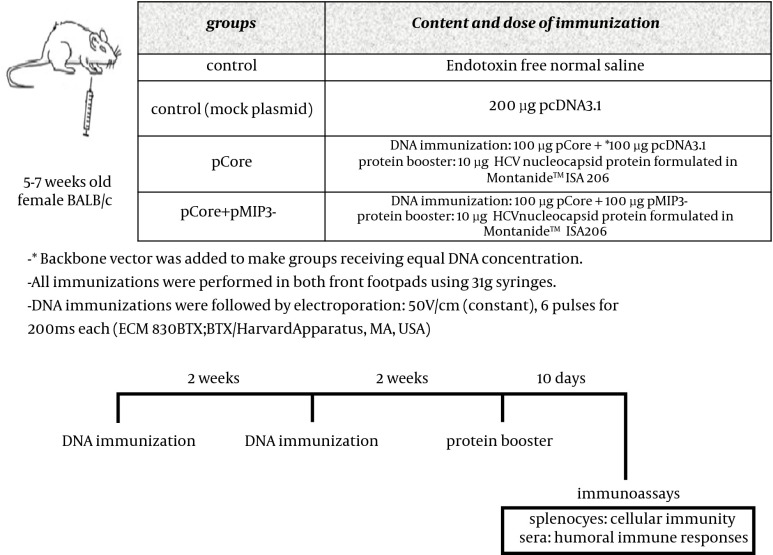
Study Design, Immunization Regimen and Description of Groups

Mice were immunized according to the schedule provided in Figure 2. Ten days after final immunization, spleens of immunized mice were isolated, smashed, the RBCs were lysed and single cell suspension was prepared by suspending splenocytes in Advanced RPMI-1640 medium containing 5% FBS, 1x GlutaMAX , 1x penicillin/streptomycin. A cocktail of peptide/protein consisting of 2 HCV core H2-D–restricted epitopes (core39-48; RRGPRLGVRA) (22) and core133-142; LMGYIPLVGA) (23) and HCV nucleocapsid protein (Abcam, The UK) was used for in vitro recall experiments.

#### 3.4.2. Lymphocyte Proliferation

Non-radioactive Cell Proliferation ELISA, BrdU (colorimetric) kit (Roche, Germany) was used to evaluate lymphoproliferative responses. 3×10^5^ cells of single cell suspension were cultured in each well of 96-well flat bottom plates, and recalled by peptide/protein cocktail for 72 h at 37°C. BrdU labeling was performed according to the manufacturer’s protocol. Anti-BrdU-POD was applied to wells followed by substrate addition to measure incorporation of BrdU into cell DNA by colorimetric analysis at A450nm. Stimulation Index (SI) for each sample was calculated as OD450nm of stimulated well / OD450nm of un-stimulated well. 

#### 3.4.3. Cytokine ELISA and ELISpot Assay

Single cell suspension in concentration of 2 × 10^6^ cells/mL was cultured in 24-well plates, stimulated with peptide/protein cocktail for 72h and supernatants were subjected to measure IFN-γ and IL-4 levels using commercial ELISA kits (R&D systems, Minneapolis, MD). Quantification of core specific IFN-γ secreting cells was performed with ELISpot assay kit (MabTech, Sweden) according to the manufacturer’s instruction. 

#### 3.4.4. Granzyme B (GrzB) Release Assay

The efficacy of immunization to develop the release of cytolitic effector GrzB molecule by immune cells after re-stimulation with cognate antigen/peptide was evaluated by mouse GrzB ELISA (R&D systems). Briefly, splenocytes were cultured in 96-well culture plates with or without peptide/protein cocktail. After 60 hrs incubation at 37°C, supernatants were collected and subjected for ELISA following manufacturer’s instructions. Core specific GrzB release was measured as follows: Specific GrzB release (pg/mL) = Experimental release (pg/mL) – Spontaneous release (pg/mL).

### 3.5. Humoral Immune Responses

Core specific IgG antibodies were analyzed by ELISA as described previously with some modifications (20). In brief, Maxisorb 96 well plates (Nunc, Denmark) were coated overnight at 4°C with 0.5 µg of nucleocapsid protein (Abcam, The UK) dissolved in 100 µL carbonate coating buffer (CBC). Wells were washed with washing buffer (0.05% Tween 20 in PBS) and blocked to inhibit non-specific protein binding with blocking buffer (3% BSA in PBS). Mice sera were applied to wells and incubated for 2 h at 37°C. After washing steps, 100 µL of 1:7000 diluted HRP-conjugated anti- mice IgG (Sigma) was applied for 1 h at 37°C. TMB (tetramethylbenzidine) was added to wells to develop colorimetric reaction, and optical density (OD) was measured at 450nm with a plate reader after the reaction was stopped by adding 2N H2SO4. To determine anti-core specific IgG subtypes, IgG1 and IgG2a subclasses were measured using goat anti-mouse IgG1 and IgG2a secondary antibodies (Sigma) according to the same protocol and manufacturer’s instructions.

### 3.6. Statistical Analysis

SPSS software version 15 (SPSS Inc., Chicago, IL, The USA) was used for statistical analysis. Data were expressed as mean ± SD. Student’s t-test or one-way ANOVA test was performed for data comparison and P value less than 0.05 was considered as statistically significant. 

## 4. Results

### 4.1. Expression and Biological Activity of MIP-3beta

The plasmid expressing MIP-3beta was verified by restriction digestion, PCR and DNA sequencing (data not shown). The expression and secretion of MIP-3beta was confirmed in supernatant of pMIP-3beta-transiently transfected HEK293T cells by using commercial sandwich ELISA kit (Figure 1 A). The ability of the expression product from pMIP-3beta to stimulate migration of CCR7 + HUT-78 cell line was evaluated by the migration transwell assay. As shown in Figure 1 B, supernatant from pMIP-3beta-transiently transfected HEK293T cells showed potent chemoattractant activity, and this MIP-3beta mediated chemotaxis was significantly decreased by the addition of anti MIP-3beta neutralizing antibody, further demonstrating the chemokine specific chemoattraction (Figure 1 C).

### 4.2. Cellular Immune Responses

**Figure 3.**
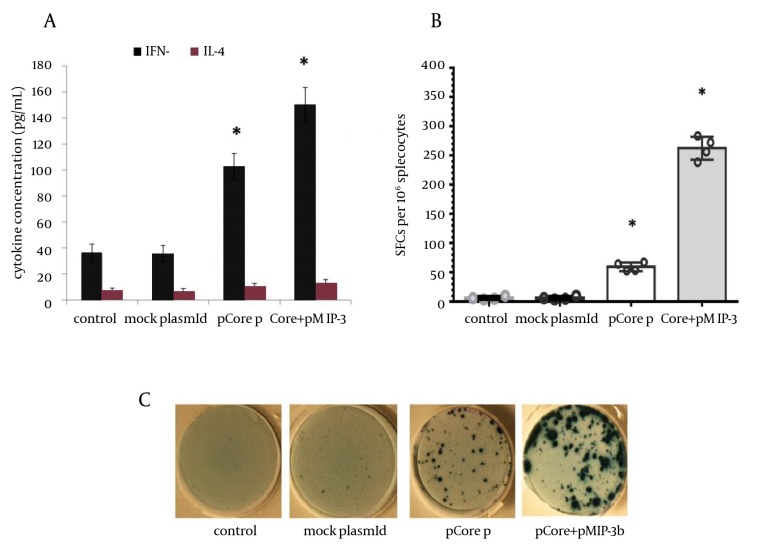
Cytokine ELISA and ELISpot Assay A) Supernatant of cultured splenocytes was collected after 72h of recall by core protein/peptide cocktail and was assessed for IFN-gamma and IL-4 by using ELISA. Data were representative of mean cytokine concentration (pg/mL) ± SD of four mice per group in triplicate. B) IFN-gamma ELISpot was performed to identify the relative population of core specific IFN-gamma producing cells. The circles represent average of SFCs /10^6^ splenocytes in triplicate assays for each mouse (n=4 mice per group), while error bars indicate SD. Asterisks indicate the significant difference among labeled groups and/or the control groups. C) Representative images of ELISpot wells of each experimental group (antigen-stimulated wells).

**Figure 4.**
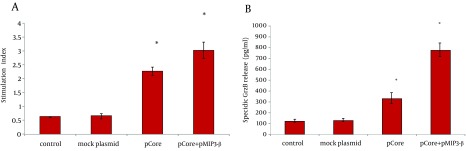
A) The average proliferation of lymphocytes after in vitro recall of each group of mice. B) Core specific cytolytic GrzB release was calculated as described in Materials and Methods. Data represents the mean ± SD of 4 mice per group in triplicate. Asterisks indicate the groups which are statistically different from each other and/or control groups.

To determine the immunomodulatory effects of MIP-3beta gene codelivery on cellular immune responses, splenocytes of the immunized mice were used for cytokine release, lymphoproliferative and cytolytic GrzB release assays. The core specific releases of IFN-γ and IL-4 by in vitro stimulated splenocytes were measured in the supernatant of cultured cells. Co-inoculation of pMIP-3beta and pCore enhanced the production of antigen/epitope specific IFN-γ 1.5 fold higher than with pCore alone group; while in all the experimental groups IL-4 release did not significantly differ from the control ones (Figure 3 A) which demonstrated an amplified Th-1 polarized response. Accordingly, results of ELISpot assay showed a drastic difference in the number of IFN-γ secreting cells in favor of mice co-vaccinated with pMIP-3beta compared to pCore alone group (262.2 ± 17 vs. 59.2 ± 6.5 of IFN-γ SFCs/10^6^ of cultured splenocytes respectively) (P < 0.01) which indicated the capability of MIP-3beta chemokine as an adjuvant to expand the number of core specific IFN-γ secreting cells (Figure 3 B). Results of lymphoproliferative responses revealed a significant antigen specific increase in number of splenocytes of mice co-vaccinated with pMIP-3beta and pCore. We previously showed that pCore immunization induced a brief lymphoproliferative response (24) and these results showed that pMIP-3-beta co-administration enhanced these responses up to 1.4 fold (P < 0.05) (Figure 4 A). Acquisition of cytolytic GrzB molecule release after restimulation with cognate antigen/peptide by immunized splenocytes was assessed with quantification of specific GrzB release amount following in vitro recall. Cytolytic GrzB release is one of the major mechanisms of cytolytic immune cells to kill infected or malignant cells. pMIP-3beta co-administration induced a significantly higher percentage of antigen/epitope specific GrzB release (772.3 ± 63.3 pg/mL) compared to the pCore group (328.4 ± 50.6 pg/mL) (P < 0.01), suggesting that this chemokine can act as an adjuvant in enhancement of cytolytic functions of immunized splenocytes (Figure 4 B). 

### 4.3. Humoral Immune Responses

**Figure 5.**
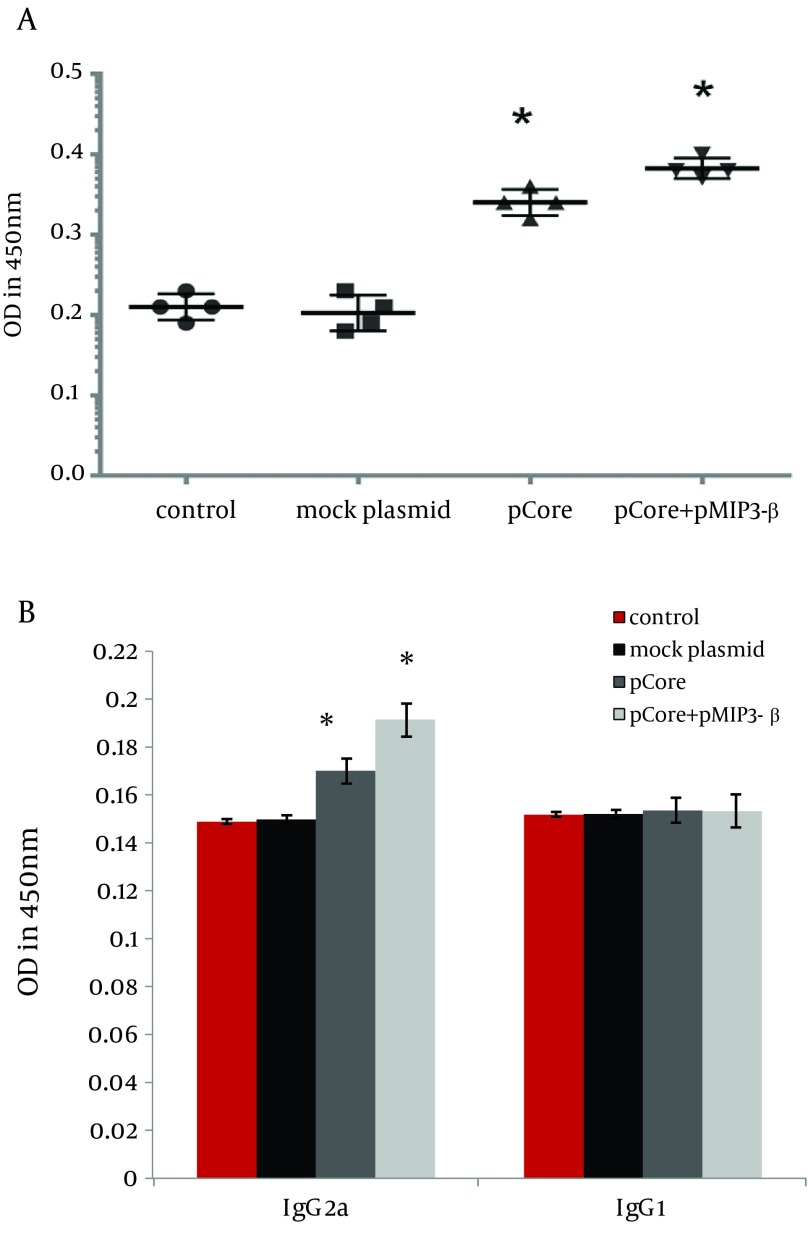
Determination of Core Specific Humoral Immune Responses ELISA was used to measure core specific total IgG (A), IgG1 and IgG2a subtypes levels (B). Data displays averages of OD absorbance at 450nm of four mice per group in duplicates ± SD. Asterisks indicate the groups showing statistically significant difference from each other and/or the control groups.

pMIP-3beta co-immunization slightly elicited total anti-core IgG level (Figure 5 A) (P < 0.01). IgG1 and IgG2a subtypes of specific IgG were evaluated as well to analysis its modulatory effects. IgG2a level was markedly increased by MIP-3beta co-administration compared to the pCore group; while the higher IgG2a/IgG1 ratio was in accordance with an augmented Th1 polarized immune response (Figure 5 B).

## 5. Discussion

A desired HCV vaccine should be able to elicit high levels of antigen specific cellular and humoral responses. DNA vaccine modalities might be suitable as - HCV vaccine candidates, but they require co-application of other strategies (such as electroporation mediated antigen delivery) and/or co-administration of immune modulators to enhance their immunogenicity (25, 26). In the current study, we evaluated the potential use of homeostatic CCL-19/MIP-3beta chemokine to modulate the immune responses of HCV core DNA vaccine. Although the potential role of molecular adjuvants like cytokines and costimulatory molecules in the vaccination strategies has been extensively investigated (10, 11), but the positive effect of chemokines as molecular adjuvants remains to be further evaluated.By virtue of its paramount importance in immune response formation and diverse effects on the DCs and lymphocyte migration and activationMIP-3beta has been postulated as an interesting candidate vaccine adjuvant for both cancer and infectious disease regarding its paramount importance in immune response formation and diverse effects on the DCs and lymphocyte migration and activation (27). Our data indicated that co-delivery of pMIP-3beta with HCV core DNA vaccine improved Th1 polarized cell-mediated immune responses evidenced by an increase in: IFN-γ/IL-4 ratio (Figure 3 A), relative number of IFN-γ producing cells (Figure 3 B), lymphocyte proliferative responses (Figure 4 A), cytolytic GrzB release activity (Figure 4 B) and the core specific IgG2a/IgG1 ratio (Figure 5 B) compared with the corresponding controls. Moreover, humoral responses (anti-core total IgG) levels were also enhanced in mice co-immunized with pMIP-3beta (Figure 5 A) compared to the corresponding controls. Our results are in accordance with a prior report on co-administration of MIP-3beta gene with a beta-gal expressing plasmid indicating that co-administered formulation enhanced both theTh1 polarized cell-mediated and humoral immune responses as well as specific CD8+ T cells activities in a tumor cell-challenge vaccine study (16). Accordingly, co-delivery of MIP-3beta gene enhanced both antigen specific total IgG and IFN-γ/IL-4 ratio of a Streptococcus mutans pac gene expressing DNA vaccine (28), while genetic co-transfer of MIP-3beta improved both Th1/Th2immune responses in HIV gp140 (28) and HSV-1 gB DNA vaccine studies (17). Subunit vaccines (formulation of recombinant antigens in proper adjuvants) are recognized as efficient and safe vaccine modalities for human immunization. In fact, the only approved recombinant vaccines for human use (against Hepatitis B and Human Papilloma viruses) are of this type (29). The results of our study on HCV core DNA vaccine co-administration with MIP-3beta, in induction of proper Th1-oriented (Figures 3, 4 and 5A) and humoral responses (Figure 5 B) are in complete agreement with outcome of Montanide (22) and Bacillus Calmette-Guerin (BCG) (30) formulated HCV core protein immunization studies in mice regarding the elicited responses. Indeed, results of our cytolytic GrzB release activity (Figures 4 B, C) is even superior to that of BCG formulated HCV core protein (30). Moreover, our results are comparable with the data of HCV core-ISCOMATRIX (formulation of HCVcp 1-191 in negatively charged immuno-stimulating complex) immunization studies in rhesus macaques and a phase-I study in human regarding the induction of both antibody responses and T-cell cytokines following vaccine administration (26). Therefore, HCV core DNA vaccine co-administration with MIP-3beta might elicit antigen specific immune responses comparable/superior to that of candidate HCV core subunit vaccines.

The cardinal immunomodulatory mechanism of MIP-3beta as vaccine adjuvant remains to be completely elucidated. The chemokine, MIP-3beta, is constitutively expressed in T cell zone of lymphoid tissues and establishes a microenvironment for interaction of mature DC, naïve T cells and B cells to initiate primary immune responses (15). It has been shown that it enters secondary lymphoid tissues and expresses the chemokine following administration of MIP-3beta plasmid (28). The distribution of plasmid DNA and its RNA transcript in lymphoid tissues following pMIP-3b administration has been demonstrated previously (28). It is assumed that exogenous MIP-3beta overexpression in lymphoid tissue may enhance the recruitment of APCs, facilitating the chance of interaction between DC, T- and B cells in lymphoid tissues and consequently enhancing both humoral and cell-mediated immune responses. This conception is supported by the observation that plt/plt knockout mice (lacking functional CCR7 ligands) show a failure in DC and lymphocyte homing and localization (31). Delayed antibody responses and defective T cell mediated immune reactions were also observed in such a mice. Furthermore, the increase in number of mature DCs and T cells in secondary lymphoid tissues following pMIP-3beta administration has been reported which further supports the aforementioned concept (28). 

In conclusion, the data presented in this study demonstrated the immunomodulatory effect of MIP-3beta to enhance core specific cell-mediated and humoral responses in HCV core DNA immunization. To our knowledge, this is the first study reporting the adjuvant activities of MIP-3beta in an HCV DNA vaccination model. 
